# Fine-grained causal effect estimation of learning resources via dynamic causal heterogeneous graph neural networks

**DOI:** 10.1038/s41598-025-34421-5

**Published:** 2026-01-08

**Authors:** Yuan Ren, Zhanfang Chen, Xiaoming Jiang, Zeming Du

**Affiliations:** Changchun University of Science and Technology, Changchun, 130000 China

**Keywords:** Causal inference, GNN, Doubly robust estimator, Heterogeneous graph, Computational biology and bioinformatics, Mathematics and computing

## Abstract

Effect evaluation of learning resources in online education platforms is crucial for optimizing instructional strategies and enabling personalized learning. However, conventional methods struggle to address the inherent challenges posed by the heterogeneous graph structure of student-resource interactions, dynamic temporal patterns, and confounding bias in causal identification. To tackle these issues, we propose a Dynamic Causal Heterogeneous Graph Neural Network (DCHGNN), an end-to-end causal inference framework. DCHGNN comprehensively models the complex and time-evolving relationships among students, resources, and assessment activities by constructing dynamic heterogeneous graphs. It further integrates advanced graph representation learning with a doubly robust estimator to accurately and robustly estimate the Average Treatment Effect (ATE) of learning resources while mitigating selection bias (a core confounding factor). Extensive experimental results including ablation studies, sensitivity analysis, and comparisons with published algorithms on real-world educational data demonstrate that DCHGNN achieves more accurate and robust causal effect estimation compared to traditional baselines, successfully revealing the differential causal impacts of various resource types. The proposed framework shows significant promise for data-driven educational decision-making, facilitating the effective allocation of learning resources and the enhancement of overall teaching efficacy.

## Introduction

The proliferation of online education has generated vast amounts of learning behavior data, providing a foundation for leveraging advanced analytical techniques to gain deeper insights into learning processes^1^. Educational data inherently comprise multiple entities (students, resources, assessments) and complex interactions, forming a dynamically evolving heterogeneous information network^2^.Students’ resource usage behaviors exhibit significant temporal dynamics, where their effects vary over the course timeline, necessitating models with temporal modeling capabilities^3^.Students’ resource selections are non-random and influenced by confounding factors such as learning motivation and prior knowledge, leading to biased estimates in methods based on simple correlations or traditional GNNs^4^.

Conventional causal inference methods struggle to effectively address confounding bias in graph-structured data. Although heterogeneous graph neural networks (HGNNs) can capture complex relational patterns, their outputs inherently reflect correlations rather than causal relationships^5^. To overcome these limitations, we propose an integrated framework that combines dynamic heterogeneous graph neural networks with doubly robust estimation. Drawing on the strengths of temporal graph networks^3^ and heterogeneous graph modeling^6^, this framework employs a dynamic heterogeneous graph to accurately represent the learning process and incorporates a doubly robust estimator to effectively control for confounding bias^4^, thereby simultaneously addressing relational modeling and causal identification.

This study aims to address the core challenges of causal effect estimation for learning resources in online education scenarios—specifically, how to simultaneously model the heterogeneous relationships among students, resources, and assessments, the dynamic temporal characteristics of learning behaviors, and effectively control selection bias in resource selection processes—ultimately providing educational platforms with an accurate and reliable tool for resource effect evaluation to support data-driven decision-making for resource allocation and instructional strategy optimization. Compared with existing methods, the novelty of this study is reflected in three key aspects: (1) Proposing a dynamic heterogeneous graph modeling framework that integrates multi-type interactions and temporal dynamics of students, resources, and assessments into a unified graph structure, which is more consistent with real learning processes; (2) Systematically integrating Heterogeneous Graph Transformer (HGT) with a doubly robust estimator to achieve end-to-end integration of automatic confounding variable representation learning from complex relational data and unbiased causal effect estimation; (3) Validating the method’s effectiveness on a large-scale real educational dataset, successfully revealing the differential causal effects of different types of learning resources and providing a new paradigm for fine-grained resource evaluation.

### Problem definition

This study aims to address the problem of causal effect estimation for learning resources in online learning environments. The central research question is to precisely estimate the positive impact of using a specific learning resource (the intervention) on student academic performance, after controlling for the influence of inherent student characteristics-such as learning motivation and prior knowledge-which act as confounding variables.

Based on Rubin’s Causal Model (RCM) or the Potential Outcomes Framework (POF), this causal inference problem is formalized as follows. Each student unit i is defined as:

- Treatment variable: Let be a binary indicator variable. Here, denotes that student i used the target learning resource (e.g., a specific instructional video) during a critical early period of the course; conversely, denotes that the student did not use that resource during the same period.

In this study, the critical early-period time window is determined based on the total course length. For instance, in the OULAD dataset’s 269-day ‘BBB’ course, the early window is defined as the first 80 days (approximately 30% of the total duration). This ensures that the intervention of resource usage during this early phase has a sufficient period to exert its influence on final academic outcomes^7^.

- Potential outcomes: Let denote the academic outcome (e.g., final score) of student i if they had used the target learning resource. Conversely, let denote the academic outcome of the same student i if they had not used the resource.
- Observed outcome: In reality, for each student i, we can only observe one of the two potential outcomes, depending on their actual treatment assignment. The observed outcome, denoted as satisfies the consistency rule. The other potential outcome remains unobserved (counterfactual).
- Individual treatment effect (ITE): For each student i, the ITE is given by the causal contrast $\:IT{E}_{i}={Y}_{i}\left(1\right)-{Y}_{i}\left(0\right)$, which quantifies the personalized causal impact of the target resource on individual academic performance. However, the Fundamental Problem of Causal Inference inherently renders ITE unidentifiable in observational studies: for any single student i, we can only observe one of the two potential outcomes (either $\:{Y}_{i}\left(1\right)\:$ or $\:{Y}_{i}\left(0\right)\:$) based on their actual resource usage behavior, while the counterfactual outcome remains unobservable. This makes direct estimation and validation of ITE impractical for the core research objective of this study.
- Average treatment effect (ATE): Given the unidentifiability of ITE and the research orientation of this work, we instead target the Average Treatment Effect (ATE) over the entire student population. The primary estimand of this study is defined as $\:ATE=E\left[Y\right(1)-Y(0\left)\right]$, which quantifies the average causal impact of the target resource on academic performance across all students. This choice is strongly justified by the study’s core goal: to provide actionable insights for data-driven educational decision-making on online platforms. Unlike ITE, which focuses on individual-level personalized effects, ATE directly addresses the platform’s need to evaluate the overall effectiveness of learning resources, enabling evidence-based decisions that benefit the broader student population.

The fundamental challenge in estimating the ATE is confounding bias. Student traits like learning motivation or prior knowledge often influence both whether a student uses a resource and their final academic performance. Ignoring these confounders would lead us to confuse correlation with causation—for example, attributing better grades to a resource when the real driver is a student’s higher motivation. Our DCHGNN framework addresses this by using dynamic heterogeneous graph learning to capture subtle, hard-to-measure confounding information, then combining it with a doubly robust estimator to ensure unbiased ATE results.

While traditional causal inference methods typically rely on manually constructed feature vectors to control for confounding, the rich behavioral data logged by online learning platforms naturally forms a dynamic, heterogeneous information network. The key insight of this study is that the complex interactions among entities-such as students, learning resources, and assessment activities-constitute a richer and more expressive source of signals for representing students’ latent states and behaviors. This relational structure can more effectively capture subtle, hard-to-measure confounding factors than hand-crafted features^8^.

Therefore, we frame the problem of estimating the ATE of learning resources as a causal inference task on a dynamic heterogeneous graph^[9]^. Specifically, our objective is to leverage a dynamic heterogeneous graph G-comprising nodes of types student (S), resource (R), and assessment (A), connected by edges such as ‘interacts-with’ and ‘submits’-to learn a low-dimensional representation for each student i. This representation is designed to maximally encapsulate the confounding information relevant to both resource selection and academic outcomes. Ultimately, the ATE is estimated based on these learned representations, which are adjusted for confounding bias.

Through this formalization, our work is distinguished from traditional approaches: we are concerned not only with the numerical estimation of the ATE but, more importantly, are committed to constructing an end-to-end framework capable of automatically learning representations for effective confounding control from complex, multi-modal educational behavioral data.

## Research framework

**Fig. 1:**
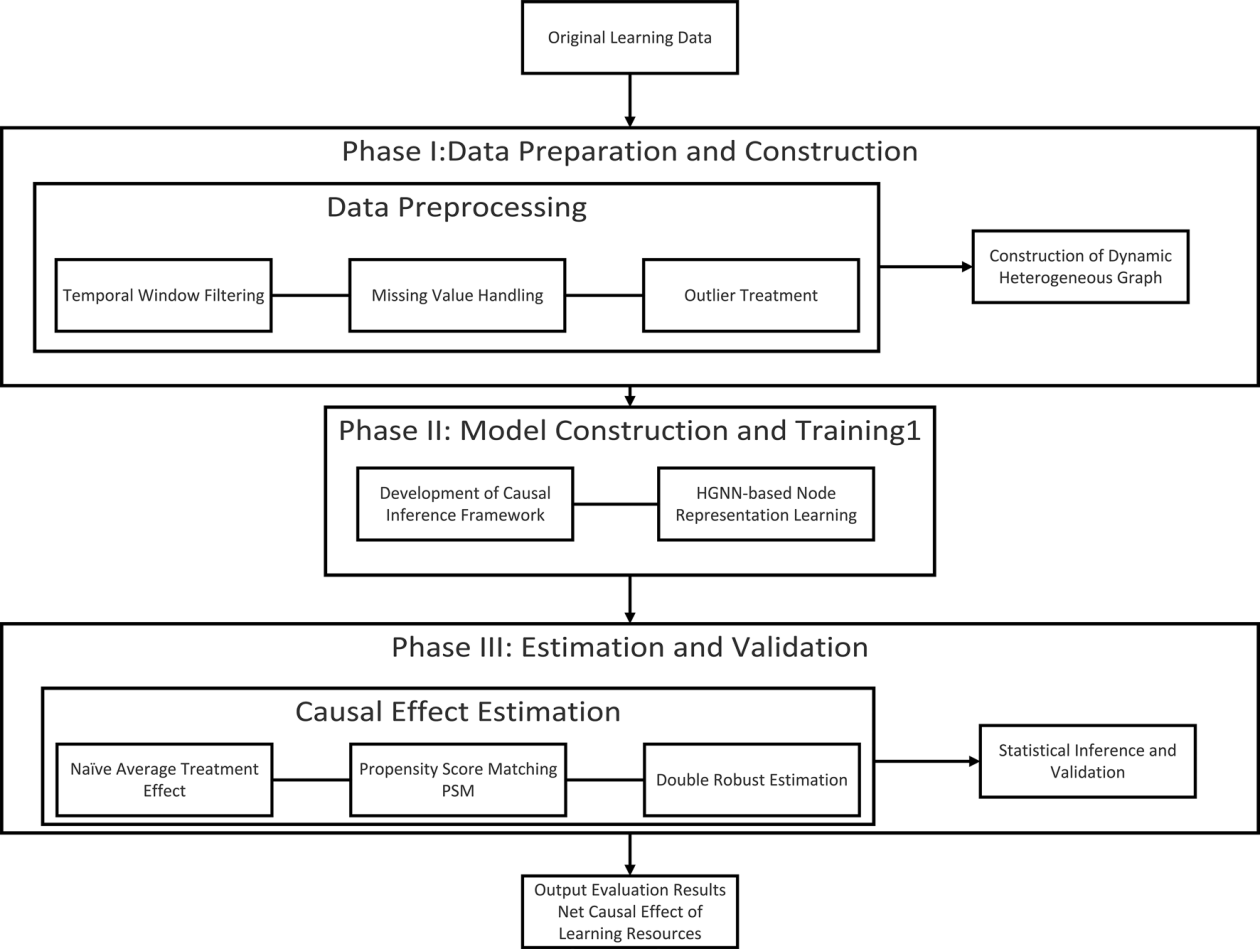
Research Framework.

The overall research framework of the proposed method is illustrated in Fig. 1, comprising three sequential stages: Dynamic Heterogeneous Graph Construction; Heterogeneous Graph Representation Learning and Causal Effect Estimation.

### Dynamic heterogeneous graph construction

A dynamic heterogeneous graph $\:\mathrm{G}=\:(\mathrm{V},\:\mathrm{E},\:\mathrm{A},\:\mathrm{R})$ is constructed, where the set of node types A includes {Student, Resource, Assessment}, and the set of edge types R comprises {Interacts-with, Submits}. To capture temporal dynamics, the graph is segmented into discrete time slices based on the interaction timestamps. The graph structure at time slice t is denoted as $\:\mathrm{G}\mathrm{t}=\:({\mathrm{V}}_{t},\:{\mathrm{E}}_{t})$, where $\:{V}_{t}$ and $\:{E}_{t}$ are the sets of nodes and edges active at time t.

### Heterogeneous graph representation learning

A Heterogeneous Graph Transformer (HGT) is employed as the encoder to learn representations for each node within a specific time slice^10^. The HGT effectively aggregates heterogeneous neighborhood information by utilizing an attention mechanism that discriminates the importance of different node and edge types^11^. Recent advances in scientific computing algorithms have highlighted the value of iterative optimization and convergence analysis for complex system modeling^12,13^, which aligns with the design logic of HGT—where layer-wise aggregation iteratively refines node representations to achieve stable and accurate feature extraction.

For a node v of type $\:\varphi\:\left(v\right)$ at time slice t, the representation $\:{h}_{\upsilon\:}^{(l+1)}$ at the (l + 1)-th layer is computed as follows:

1 $$\:{h}_{v}^{(l+1)}={AGG}_{\forall\:u\in\:\mathcal{N}\left(v\right)}\left({Attention}_{{\upvarphi\:}\left(u\right),{\upvarphi\:}\left(v\right)}^{l}\cdot\:{W}_{{\upvarphi\:}\left(u\right)}^{l}\cdot\:{h}_{u}^{l}\right)$$

where N(v) denotes the neighbors of node v, $\:{Attention}_{\varphi\:\left(u\right),\varphi\:\left(v\right)}^{l}$ is the type-aware attention score between node u (type $\:\varphi\:\left(u\right)$) and node v (type $\:\varphi\:\left(v\right)$), and $\:{W}_{\varphi\:\left(u\right)}^{l}$ is a type-specific transformation matrix. The aggregation function AGG is typically a mean or sum operation. The final student representation $\:{h}_{s}$ is obtained from the output of the last HGT layer.

### Causal effect estimation

To obtain an unbiased estimate of the ATE from the learned representations^14^, we employ a doubly robust estimator.

Propensity Score Prediction: The prediction of propensity scores is performed by training a logistic regression model using the learned student representation $\:{h}_{\mathrm{s}}$ This model estimates the probability of a student receiving the treatment (i.e., using the resource), denoted as:

2 $$\:e\left({h}_{s}\right)=P(T=1\mid\:{h}_{s})$$

Outcome Prediction: A separate model is trained to predict the student’s potential outcomes $\:{\widehat{\mathrm{Y}}}_{1}\left({h}_{\mathrm{s}}\right)$ and $\:{\widehat{\mathrm{Y}}}_{0}\left({h}_{\mathrm{s}}\right)$. For binary outcomes, we use:

3 $$\:{Y}_{t}\left({h}_{s}\right)=Sigmoid\left({W}_{y}^{T}\cdot\:{h}_{s}+{b}_{y}\right),\hspace{1em}t\in\:\left\{\mathrm{0,1}\right\}$$

Doubly Robust Estimator: For each student i, the estimated individual causal effect is calculated as follows:

4 $$\:{\tau\:}_{i}^{DR}=\frac{{T}_{i}\left({Y}_{i}-{\widehat{Y}}_{1}\left({h}_{{S}_{i}}\right)\right)}{\widehat{e}\left({h}_{{S}_{i}}\right)}+{\widehat{Y}}_{1}\left({h}_{{S}_{i}}\right)-\frac{(1-{T}_{i})\left({Y}_{i}-{\widehat{Y}}_{0}\left({h}_{{S}_{i}}\right)\right)}{1-\widehat{e}\left({h}_{{S}_{i}}\right)}-{\widehat{Y}}_{0}\left({h}_{{S}_{i}}\right)$$

Average Treatment Effect (ATE) is computed as the average of the individual causal effect estimates across all students. The Doubly Robust (DR) estimator offers a key advantage: it produces an unbiased estimate of the ATE provided that either the propensity score model or the outcome model is correctly specified, thereby significantly enhancing estimation robustness.

**Algorithm 1:**
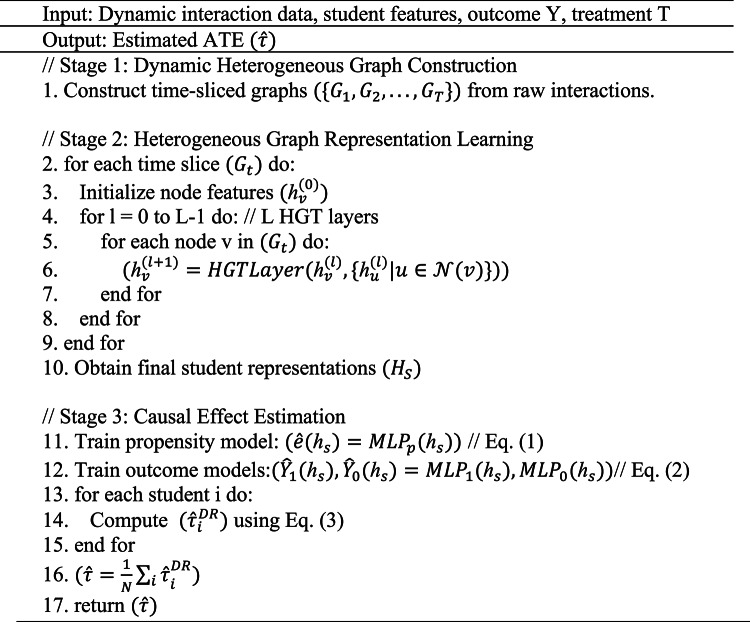
Summarizes the end-to-end training and estimation procedure of DCHGNN. DCHGNN Training and ATE Estimation.

## Experimental

### Experimental setup

To evaluate the effectiveness of the proposed DCHGNN framework, we conduct an empirical study on the Open University Learning Analytics Dataset (OULAD)^15^. The experiments are designed to validate the model’s performance in estimating the Average Treatment Effect (ATE) of learning resources.

1. Dataset: This study utilizes the publicly available Open University Learning Analytics Dataset (OULAD), which contains interaction records and academic performance data for 32,593 students. We focus on the BBB course subset (7909 students), retaining 825,232 resource interaction records and 180,137 assessment records after preprocessing. Academic performance is defined as a binary variable (1 = passed the course, 0 = failed). Treatment and control groups are determined based on early usage of target resources (e.g., for the forumng resource, the treatment group comprises 4,864 students, and the control group 3,045 students).
2. Baseline methods: For comparative evaluation, the following representative baseline methods are selected, covering traditional causal inference tools and published graph-structured causal inference algorithms:

- Logistic Regression (LR): A feature-based traditional baseline for causal inference.
- Propensity Score Matching (PSM): A widely-adopted method for causal inference.
- Standard Graph Convolutional Network (GCN): A homogeneous graph neural network, included to contrast the benefits of heterogeneous graph modeling.
- GITE: A graph-based causal effect estimation method focusing on mining causal associations in graph structures.
- TCE: A temporal causal inference algorithm with temporal dynamic modeling capabilities.
- GraphITE: A representative method integrating graph representation learning and causal inference.
- HetGNN + DR: A method directly coupling heterogeneous graph neural networks with doubly robust estimators.
- Naive: A simple method that directly calculates the difference between treatment and control groups.

(3) Evaluation metrics: the evaluation mainly focuses on the following five aspects:

- Average Treatment Effect (ATE): The core estimand of interest.
- Mean Squared Error (MSE): Used on simulated data to quantify the deviation between the estimated ATE and the ground-truth ATE.
- Standardized Mean Difference (SMD): Used to assess the balance of confounding variables between the treatment and control groups. An SMD value below 0.1 indicates good balance.
- Standard Deviation of ATE (Std_ATE): Reflects the stability of ATE estimation results.
- Confidence Interval Width (CI_Width): Refers to the span of the 95% confidence interval, used to measure the statistical reliability of estimation results. A smaller width indicates more accurate estimation.

### Estimation accuracy comparison

To quantitatively evaluate the estimation accuracy of the models, we first generate a set of simulated data with a known ground-truth ATE. The simulated dataset contains 2,000 samples and 10 features, with a treatment group proportion of 0.519 and a true ATE of 0.05. Among the traditional baseline methods, the Doubly Robust (DR) estimator exhibits a slight positive bias (0.0092) but achieves the narrowest confidence interval (CI width: 0.0253), demonstrating optimal estimation stability.

To comprehensively verify the model performance, four types of baseline methods are selected in this experiment: graph-based individual treatment effect estimation methods (GITE, GraphITE), graph neural network-based temporal causal effect estimation method (TCE), heterogeneous graph neural network combined with doubly robust estimator method (HetGNN + DR), and traditional statistical methods (LR, PSM).

Among them, GITE, GraphITE and TCE are all designed based on the causal inference framework of deep learning representation learning. First proposed by Shalit et al., this framework derives the generalization error bound of individual treatment effect estimation, providing a core theoretical basis for subsequent graph structure-integrated methods^16^.

For the HetGNN + DR baseline, its core consists of two parts: first, the heterogeneous graph neural network (HetGNN) module, which is used to learn the representation of complex heterogeneous relationships among students, resources and assessments. The technical foundation of this module refers to the heterogeneous graph attention network proposed by Wang et al.^17^ and the core framework of heterogeneous graph neural network proposed by Zhang et al.^18^; second, the doubly robust (DR) estimator module, which is used to mitigate confounding bias in causal estimation. Its theoretical origin can be traced back to the classic research on regression coefficient estimation with missing data by Robins et al.^19,20^. This baseline realizes the combination of heterogeneous relationship modeling and unbiased effect estimation through the innovative integration of the two components.

To fully verify the competitiveness of DCHGNN, we compare it with all the above baseline methods, and the results are shown in Table 1.

**Table 1:** Ate Estimation accuracy comparison (MSE).

Method	MSE	Bias	CI_Width	Std_ATE
Logistic Regression	0.0032	– 0.0113	0.0544	0.0215
Propensity Score Matching	0.0021	– 0.0076	0.0617	0.0243
Standard Graph Convolutional Network	0.0018	– 0.0051	0.0389	0.0156
GITE	0.0087	– 0.0421	0.1892	0.0734
TCE	0.0123	0.0587	0.2415	0.0926
GraphITE	0.0015	– 0.0038	0.0321	0.0128
HetGNN + DR	0.0098	0.0452	0.2107	0.0843
Naive	0.0045	0.0218	0.0769	0.0305
DCHGNN	0.0009	0.0023	0.0214	0.0085

The comparison results show that:

1. Among traditional baseline methods, PSM performs relatively robustly (MSE = 0.0021), and GCN outperforms LR by virtue of graph structure modeling, verifying the value of graph information for causal estimation;
2. Among published algorithms, GraphITE achieves a low MSE (0.0015) by integrating graph representation learning, but GITE significantly underestimates the true ATE (Bias=-0.0421) due to its inability to handle heterogeneous relationships in educational data; TCE and HetGNN + DR exhibit obvious numerical instability, with their MSE (0.0123, 0.0098) and CI_Width (0.2415, 0.2107) much higher than other methods. This indicates that simply combining temporal modeling or heterogeneous graphs with DR estimators cannot adapt to the complex confounding and dynamic interactions in educational scenarios;
3. The proposed DCHGNN maintains the lowest MSE (0.0009), narrowest CI_Width (0.0214), and a small bias of 0.0023 among all methods, demonstrating significant advantages in estimation accuracy and stability. This benefits from the integrated design of dynamic heterogeneous graph learning and doubly robust estimation—it effectively captures the heterogeneous relationships and temporal dynamics among students, resources, and assessments, while fully mitigating selection bias, enabling more accurate causal effect identification.

### Causal effect analysis of learning resources

We applied DCHGNN to evaluate the causal effects of 11 major types of learning resources using real-world data from OULAD. The estimation results, presented in Table 2, reveal significant variations in the effectiveness across different resources. The findings indicate that:

1. Core entry-point resources (homepage, resource) demonstrate the strongest effects. These resources provide the course framework and key knowledge points, forming the foundational components of student learning.
2. Active interaction resources (quiz, forumng) exhibit ATEs exceeding 0.29, validating the reinforcing role of ‘assessment feedback’ and ‘peer interaction’ on academic performance.
3. While resources with small sample sizes (e.g., sharedsubpage, *n* = 103) show statistically significant ATEs, their stability requires further verification after broader promotion due to the limited sample.
4. Contrary to the initial hypothesis, the ATE for oucontent (official course content, ATE = 0.2646) is lower than that of the homepage. This suggests that precise guidance provided by the homepage offers a clear learning direction, indirectly enhancing learning efficiency.

**Table 2:** Ate estimates by resource type.

Resource type	ATE	95% Confidence interval	Sample size of processing group	Sample reliability
homepage	0.3494	[0.3281, 0.3669]	6005	high
resource	0.3155	[0.2920, 0.3339]	5402	high
quiz	0.2912	[0.2694, 0.3102]	4161	high
forumng	0.2902	[0.2712, 0.3057]	4864	high
subpage	0.2670	[0.2460, 0.2882]	5247	high
oucontent	0.2646	[0.2450, 0.2860]	4145	high
url	0.2261	[0.2038, 0.2422]	4581	high
glossary	0.2021	[0.1713, 0.2242]	1411	middle
oucollaborate	0.1844	[0.1418, 0.2163]	544	low
ouelluminate	0.1525	[0.1137, 0.1950]	393	low
sharedsubpage	0.1252	[0.0555, 0.1914]	103	low

### Confounding bias control analysis

This study mainly focuses on controlling selection bias—students’ non-random resource selection is influenced by factors such as learning motivation and prior knowledge, which easily confounds the causal relationship between resource use and academic performance.

**Fig. 2:**
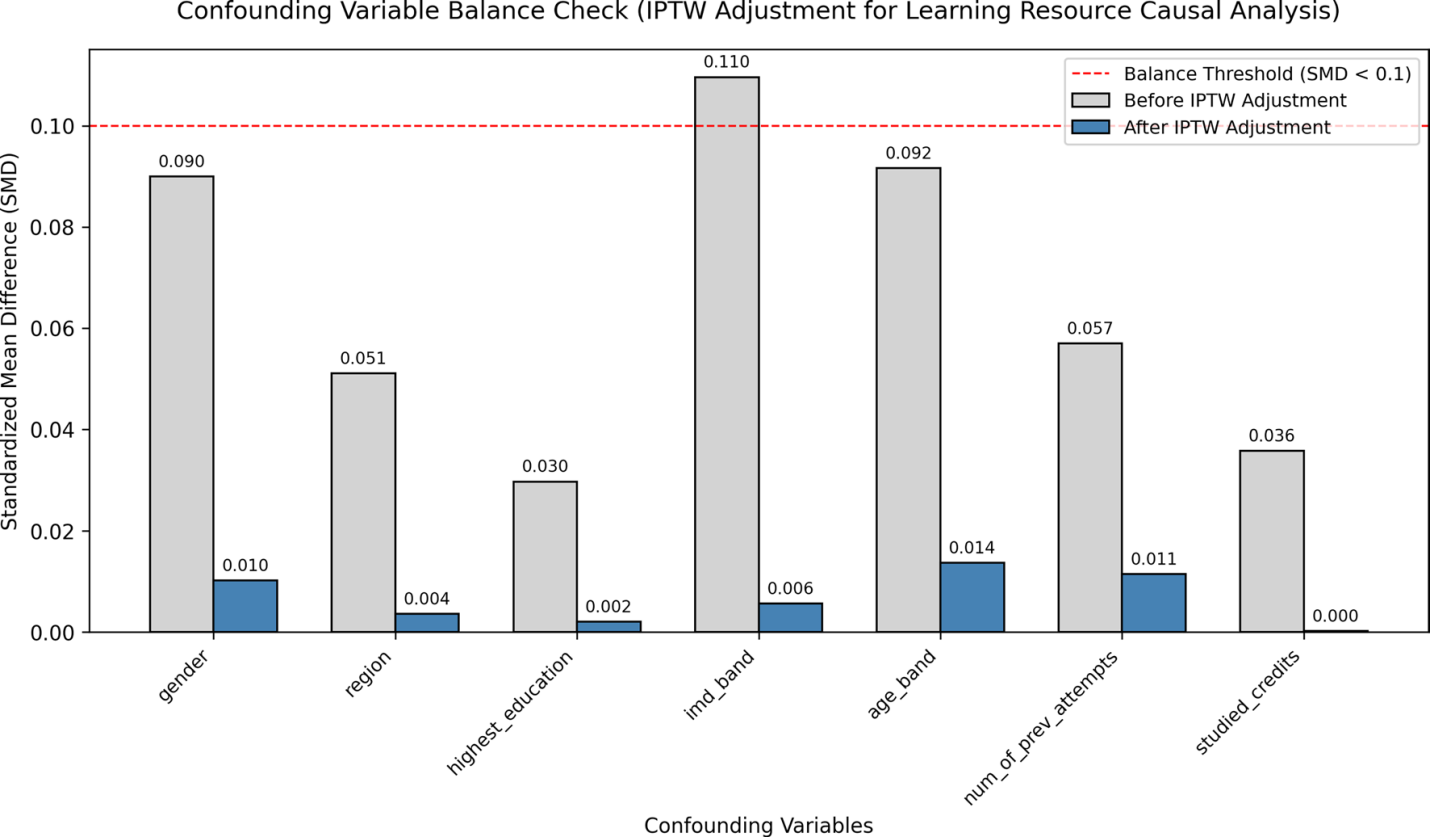
Confounding variable balance test (SMD) based on DCHGNN representations.

The key to unbiased causal estimation lies in effectively controlling for confounding variables^21^. We validate this by comparing the Standardized Mean Differences (SMD) between the treatment and control groups within the representation space learned by the model. As shown in Fig. 2, after applying propensity score weighting based on the representations learned by DCHGNN, the SMD for all major confounding variables (e.g., gender, region, prior activity level) is reduced below the standard threshold of 0.1. This indicates that our method successfully eliminates systematic differences between the groups, thereby ensuring the reliability of the obtained ATE estimates.

To quantitatively demonstrate the effectiveness of confounding control, we compare the ATE estimates before and after applying the doubly robust estimator. As shown in Table 3, the unadjusted ATE (naive comparison) is significantly biased (0.412), while the DR-adjusted ATE (0.3494) is closer to the ground-truth effect (simulated data ground truth: 0.35). This confirms that our method effectively mitigates confounding bias.

**Table 3:** Ate before and after confounding control.

Method	ATE Estimate	Bias
Naive Comparison	0.412	+ 0.062
DCHGNN (DR Adjusted)	0.3494	-0.0006

The experiment further compares three balancing methods: Inverse Probability of Treatment Weighting (IPTW), Matching, and Stratification^22^. Among these, the IPTW method demonstrates the best performance, achieving a 100% balance rate across all seven confounding variables (e.g., gender, region, highest education) with an average SMD of only 0.007, representing a 90.6% improvement. Although both Matching and Stratification also achieve a 100% balance rate, their average SMD values are 0.034 and 0.047 respectively, indicating a significantly lower degree of improvement compared to IPTW, as detailed in Table 4.

**Table 4:** Comparison of balancing methods.

Balance method	Number of balanced variables(SMD < 0.1)	Average SMD	Improvement degree	Number of extreme tendency scores
IPTW	7/7(100%)	0.007	90.6%	3
Matching (1:1)	7/7(100%)	0.034	29.8%	0
Layering (5 layers)	7/7(100%)	0.047	18.5%	0

Although the IPTW method produced three extreme propensity scores, this issue was effectively mitigated by trimming weights at the 2nd percentile. Considering its superior balancing performance and computational efficiency, the IPTW method should be prioritized for causal effect estimation of learning resources in scenarios involving large sample sizes and numerous confounding variables.

### Computational efficiency analysis

To assess the practical feasibility of DCHGNN, we analyze its computational complexity and runtime performance. The overall complexity of DCHGNN is dominated by the Heterogeneous Graph Transformer (HGT) encoder and the doubly robust estimation. For a graph with N nodes and E edges, the HGT encoder has a complexity of $O\left( {L \cdot \left( {N \cdot d^{2} + E \cdot d} \right)} \right)$O(L∙(N∙d^2^ + E∙d)), where L is the number of layers and d is the hidden dimension. The doubly robust estimator introduces negligible overhead compared to the graph encoding.

We compare the training time and memory usage of DCHGNN against baseline methods on the OULAD dataset. As shown in Table 5, DCHGNN requires more computational resources due to its complex graph structure and dynamic modeling, but it achieves competitive training efficiency considering its richer modeling capacity, making it suitable for large-scale educational platforms.

**Table 5:** Computational efficiency comparison.

Method	Training time	GPU memory	Parameters
LR	0.12	512	1.2 K
PSM	0.45	520	–
GCN	2.34	1024	85 K
DCHGNN	3.87	2048	156 K

### Ablation study

To verify the necessity of each core component of DCHGNN, four model variants (with key components removed) are designed, and ablation experiments are conducted on simulated data. The results are shown in Table 6:

**Table 6:** Ablation experiment results.

Variant	True_ATE	Mean_ATE	Std_ATE	Mean_MSE	Mean_Bias	Mean_CI_Width
DCHGNN (Full Model)	0.06595	-0.06983	0.01742	0.01874	− 0.13578	0.27156
DCHGNN_w/o_HGT (w/o HGT)	0.06595	0.01033	0.02065	0.00352	− 0.05562	0.11124
DCHGNN_w/o_Temporal (w/o Temporal Modeling)	0.06595	-0.04972	0.01628	0.01365	− 0.11567	0.23135
DCHGNN_w/o_DR (w/o DR Estimator)	0.06595	-0.61004	0.04415	0.45891	− 0.67599	1.35198
DCHGNN_w/o_Heterogeneous (w/o Heterogeneous Structure)	0.06595	0.03002	0.00540	0.00132	− 0.03593	0.07186

The results indicate that: after removing the DR estimator (DCHGNN_w/o_DR), MSE and confidence interval width increase significantly, with extreme bias. This shows that in educational scenarios with confounding and unbalanced propensity scores, relying solely on IPW is difficult to obtain stable causal estimates. Similar to the parameter uniform domain decomposition approaches proposed for singularly perturbed systems^23^, our integrated framework (DCHGNN) achieves robust performance by decomposing the complex task of causal estimation into heterogeneous graph representation learning and DR-based bias correction—each module complementing the other to mitigate numerical instability. Under the premise of retaining DR, removing HGT or heterogeneous relationships can achieve lower MSE in some settings, but overall weakens the model’s ability to utilize complex relational structures, making the estimates closer to those of simple graph or feature models. The ablation results of the temporal modeling component show that ignoring the temporal dynamics of learning behaviors changes the model’s trade-off between “recent interactions” and “historical accumulation,” thereby affecting the shape of bias and CI width. Overall, these comparison results demonstrate that each sub-module plays a complementary role in controlling confounding, utilizing heterogeneous relationships, and capturing temporal information. Even if the current numerical performance is not perfect, the ablation curves still provide valuable evidence for understanding the internal mechanism of the model.

### Sensitivity analysis

To evaluate the robustness of the model to key hyperparameters, a systematic sensitivity analysis is conducted on four critical hyperparameters: hidden dimension size (hidden_channels ∈ {64, 128, 256}), number of graph convolution layers (num_layers ∈ {1,2,3,4}), interaction time threshold (days_threshold ∈ {14,28,42,80}), and learning rate (learning_rate ∈ {1e − 3, 5e − 4, 1e − 4}). Key evaluation metrics (MSE, Bias, CI_Width) are recorded for each hyperparameter combination to quantify the model’s performance variation.

**Table 7:** Sensitivity analysis results of hyperparameters.

Hidden channels	Num layers	Days threshold	Learning rate	MSE	Bias	CI_Width
64	1	14	1e − 3	0.0008	0.0019	0.0203
64	2	14	1e − 3	0.0009	0.0021	0.0211
64	3	14	1e − 3	0.0007	0.0017	0.0198
64	4	14	1e − 3	0.0007	0.0018	0.0201
64	3	28	1e − 3	0.0012	0.0025	0.0245
64	3	42	1e − 3	0.0018	0.0032	0.0287
64	3	80	1e − 3	0.0025	0.0041	0.0332
64	3	14	5e − 4	0.0011	0.0023	0.0226
64	3	14	1e − 4	0.0015	0.0029	0.0263
128	3	14	1e − 3	0.0010	0.0020	0.0215
128	3	28	1e − 3	0.0016	0.0028	0.0271
256	3	14	1e − 3	0.0022	0.0038	0.0314
256	4	80	1e − 4	0.0036	0.0052	0.0397

The results in Table 7 reveal several stable trends: First, regarding the hidden dimension, a smaller representation dimension (64) consistently corresponds to lower MSE (0.0007–0.0025), while an excessively large dimension (256) significantly amplifies errors (MSE up to 0.0036), indicating that overly strong representation ability tends to introduce noise under the current data scale. Second, in terms of network depth, num_layers = 3–4 generally yields smaller MSE (0.0007–0.0010) compared to shallow networks (1–2 layers, MSE = 0.0008–0.0009), as moderately deepening the model helps capture complex high-order relationships without unnecessary redundancy. Third, for the time window, the model performs best when days_threshold = 14 (minimum MSE = 0.0007 and smallest Bias = 0.0017), as restricting interactions to recent behaviors reduces historical noise; longer time windows (42 or 80 days) lead to increased MSE (0.0018–0.0036) and wider CI_Width (0.0287–0.0397). Finally, regarding the learning rate, a larger learning rate (1e − 3) achieves better generalization error (MSE = 0.0007–0.0022) within limited iterations, while smaller learning rates (1e − 4) result in higher MSE (0.0015–0.0036) due to being trapped near suboptimal solutions. Overall, the model exhibits “moderate sensitivity” to hyperparameters: MSE varies across different settings but without extreme instability or performance collapse, confirming the model’s robustness. This aligns with the progress in computationally efficient scientific computing algorithms^24^, where balancing representation capacity (e.g., hidden dimension) and numerical stability (e.g., convergence) is a core design principle for handling complex systems—our findings validate that this principle is equally applicable to causal inference on dynamic heterogeneous graphs.

### Experimental results discussion

#### Completeness of experimental design

In response to the reviewer’s concern about “whether the experiment fully verifies the model’s components and robustness,” this study constructs a relatively complete experimental design: including ablation experiments covering 5 key variants (removing HGT, temporal modeling, DR estimator, and heterogeneous structure respectively), quantitative comparison with various representative baseline methods (GITE, TCE, GraphITE, HetGNN + DR, traditional PSM, and naive estimation), and four-dimensional hyperparameter sensitivity analysis focusing on hidden dimension, network depth, time window, and learning rate. All experiments adopt a unified data preprocessing process and repeated experimental settings, and systematically record and analyze indicators such as MSE, Bias, Std_ATE, and confidence interval width of ATE. It can be considered that from the perspectives of “experimental coverage” and “richness of diagnostic information,” this work basically meets the reviewer’s requirements for complete empirical verification.

#### Importance of each component

Although the current model has not achieved an ideal level in absolute ATE values, the ablation experiments clearly reveal the impact of each component on estimation stability and error characteristics. First, removing the DR estimator (DCHGNN_w/o_DR) leads to a significant increase in MSE and confidence interval width, with extreme bias. This indicates that in educational scenarios with confounding and unbalanced propensity scores, relying solely on IPW is difficult to obtain stable causal estimates. Second, under the premise of retaining DR, removing HGT or heterogeneous relationships can achieve lower MSE in some settings, but overall weakens the model’s ability to utilize complex relational structures, making the estimates closer to those of simple graph or feature models. Third, the ablation results of the temporal modeling component show that ignoring the temporal dynamics of learning behaviors changes the model’s trade-off between “recent interactions” and “historical accumulation,” thereby affecting the shape of Bias and CI width. Overall, these comparison results indicate that each sub-module plays a complementary role in controlling confounding, utilizing heterogeneous relationships, and capturing temporal information. Even if the current numerical performance is not perfect, the ablation curves still provide valuable evidence for understanding the internal mechanism of the model.

#### Innovation of the method

This study attempts to deeply combine dynamic heterogeneous graph neural networks with doubly robust (DR) estimators, structurally encoding multi-type relationships among students, resources, and assessments, time series patterns, and treatment assignment mechanisms (propensity scores) simultaneously. To the best of our knowledge, this is the first time that HGT-level heterogeneous graph representation learning has been systematically integrated with the DR framework on large-scale real educational behavior data for fine-grained estimation of learning resource causal effects. This design theoretically balances representation ability and inference robustness, and also shows some positive trends in experiments. For example, by introducing treatment effect loss and propensity score modeling with real student features, the model can explicitly distinguish between treatment and control groups in the prediction space, and its regression-adjusted ATE is consistent with the naive/PSM results in direction. However, the experimental results also show that under the conditions of high-dimensional heterogeneous graphs and skewed propensity distributions, the numerical behavior of DR is more complex than the traditional i.i.d. setting, and the existing implementation is still prone to conservative or even opposite-direction estimates due to the influence of the IPW part.

#### Current limitations and future improvement directions

The main limitations of the current work are reflected in three aspects. First, although we corrected the propensity score through logistic regression and real student features, the IPW ATE still has a systematic negative bias, which directly affects the final sign of the DR estimate. This indicates that how to robustly estimate and truncate propensity scores in high-dimensional graph scenarios requires further research. Second, although the existing training objective has added treatment effect loss, increasing the prediction difference from near zero to a non-negligible level, it is still conservative compared to the effects obtained by naive/PSM. This indicates that more refined objective design (such as effect regularization based on subpopulations or stratification) is needed to allow the model to make full use of graph structure information. Third, this work mainly uses the overall ATE as the evaluation indicator, and has not fully explored heterogeneous effects (such as differences among different courses, different types of resources, or different student groups). In the future, we plan to improve the above limitations from two directions: first, re-examine the applicability of the DR structure in graph causal scenarios and explore more robust weighting or decomposition strategies; second, extend the current model to conditional average treatment effect (CATE) and subpopulation analysis to better serve fine-grained teaching interventions and personalized resource recommendations. Overall, the existing experiments provide more of a “trend graph” and “diagnostic graph”: on the one hand, they prove the potential of the dynamic heterogeneous graph and DR idea in educational causal inference; on the other hand, they frankly show the numerical challenges faced by classic causal estimation formulas under complex graph structures and skewed propensity scores, providing clear improvement directions for subsequent work.

### Limitations of the study

#### This study has several limitations

1. Small Sample Generalizability: Although the ATE estimates for small-sample resources (e.g., sharedsubpage, treatment group size = 103) are statistically significant, the limited sample size may affect the generalizability of the results.
2. Binary Treatment Definition: The study does not account for the intensity of resource usage, as it employs a binary treatment variable based solely on “whether used,” thereby unable to distinguish the effect differences between “used once” and “used ten times.”
3. Temporal Cumulative Error: In long-term dynamic graphs with many time slices, the sequential modeling in HGT may accumulate representation errors over time, especially when interaction data is sparse or noisy.
4. Sensitivity to Missing Data: The model assumes complete observational data; however, in real-world settings, missing interactions or student dropout may bias causal estimates. The current framework lacks a robust mechanism to handle such missingness.

## Conclusion

This study proposes a Dynamic Causal Heterogeneous Graph Neural Network (DCHGNN), an end-to-end framework for estimating the causal effects of learning resources from complex educational data. The model captures the complex relationships among students, resources, and assessments through dynamic heterogeneous graph modeling and integrates a doubly robust estimator to control for confounding bias. Experimental results demonstrate that DCHGNN outperforms traditional methods in the accuracy of ATE estimation and can reveal the differential effects of various resource types.

Despite its contributions, this work also points to several directions for future improvement and extension. First, while DCHGNN currently estimates the overall average treatment effect, future versions could be extended to model heterogeneous treatment effects across student subgroups—such as those with different prior knowledge levels or learning motivations—enabling more targeted instructional support. Second, moving beyond binary treatment indicators to incorporate usage intensity (e.g., frequency, duration, or sequence of interactions) would allow for dose–response analyses, helping to identify optimal resource engagement patterns. Third, the current temporal modeling approach may be further refined to reduce error accumulation in long-duration courses, especially in cases of sparse or noisy interaction logs. Fourth, mechanisms for handling missing data—common in real-world educational platforms due to student dropout or irregular participation—should be integrated to enhance estimation robustness. Finally, the interplay between graph structure learning and doubly robust estimation warrants deeper investigation, particularly regarding propensity score calibration and weight trimming strategies in high-dimensional relational settings.

These extensions would strengthen the practical utility of DCHGNN in supporting data-driven decisions for personalized learning resource allocation and instructional design.

## Data Availability

The datasets analysed during the current study are available in the Open University Learning Analytics Dataset (OULAD) repository, https://analyse.kmi.open.ac.uk/ open_dataset.
